# Editorial: Neurorights and Mental Freedom: Emerging Challenges to Debates on Human Dignity and Neurotechnologies

**DOI:** 10.3389/fnhum.2021.823570

**Published:** 2021-12-22

**Authors:** Eric García-López, José M. Muñoz, Roberto Andorno

**Affiliations:** ^1^Instituto Nacional de Ciencias Penales, Mexico City, Mexico; ^2^Mind-Brain Group, Institute for Culture and Society, University of Navarra, Pamplona, Spain; ^3^International Center for Neuroscience and Ethics, Tatiana Foundation, Madrid, Spain; ^4^Asociación Mexicana de Neuroética, Mexico City, Mexico; ^5^Faculty of Law and Institute of Biomedical Ethics and History of Medicine, University of Zurich, Zürich, Switzerland

**Keywords:** neurotechnology, artificial intelligence (AI), human dignity, mental freedom, neurorights

The past three decades have witnessed extraordinary developments in neuroscience and neurotechnologies. These advances give us new insights into the brain's functioning and how it correlates to behavior. By allowing direct access to mental data and new forms of intervention in the brain, such developments have great potential to improve the wellbeing of patients suffering from neurological disorders. Moreover, the convergence of artificial intelligence (AI) technology with data from brain activity is accelerating our understanding of the mental processes underpinning human behavior. The new knowledge resulting from the confluence of neuroscience and AI and the tools derived from it are very promising in terms of the development of new diagnostic, preventive, or therapeutic measures for neurological conditions.

However, these same technologies, in particular brain-computer interfaces (BCI) and those allowing the acquisition and interpretation of brain data, create unprecedented challenges to basic human goods, such as the privacy of our mental life, freedom of thought, freedom from discrimination, freedom from self-incrimination and self-determination, just to mention a few examples. Ultimately, these developments could directly jeopardize human dignity, as they relate to the heart of human personhood and identity.

The key question of this Research Topic could be expressed as follows: *How can we take advantage of the therapeutic and diagnostic promises of neurotechnologies and AI without putting human dignity and human rights at risk?* In other words, how can we make these technological advances compatible with what we may call “mental freedom,” thus including values such as personal identity, mental privacy, cognitive liberty, psychological continuity, mental integrity, and others? What would be the appropriate policies to guarantee this compatibility? How should lawmakers and international human rights bodies respond to these challenges? More specifically, do we need to develop new human rights in this area? Or, more modestly, should lawmakers rather expand existing rights in order to cover the new range of issues posed by neurotechnologies? This Research Topic aims precisely to discuss these difficult questions by bringing together researchers from relevant disciplines including philosophy, neuroethics, psychology, law, and neuroscience.

Ienca is one of the very pioneers of the “neurorights” concept together with Roberto Andorno (see Ienca and Andorno, 2017) and the Neurotechnology Ethics Taskforce (aka Morningside Group) led by Rafael Yuste (see Yuste et al., 2017, 2021; Goering et al., 2021; NeuroRights Foundation, 2021). In his paper, he carries out an extensive study of this concept and its applications, which includes the past—how neurorights arose and how they are related to their historical antecedents—, the present—how neurorights are embedded in a conceptual taxonomy, and what efforts and initiatives are currently committed to their implementation—, and the future—what academic, ethical-legal, and policy-making challenges are still to be resolved. Ienca also proposes a complete formal definition of neurorights, which he conceives “as the ethical, legal, social, or natural principles of freedom or entitlement related to a person's cerebral and mental domain; that is, the fundamental normative rules for the protection and preservation of the human brain and mind.”

Schleim's article focuses on the relationship between the past and present of neurorights, for which he looks into the experiments carried out by José M. Rodríguez Delgado and Elliot S. Valenstein—two great pioneers of brain stimulation in the 20th century—, and into how they both faced the ethical-legal and social challenges that arose from their work. Schleim then approaches the relationship between this early neuroethics of brain stimulation and current proposals for neurorights, and ends with a conceptual analysis of the reach and limitations of neurotechnology using two current examples of BCI.

Among all the neurorights, the only one in which Ienca and Andorno (2017) and Yuste (see NeuroRights Foundation, 2021) fully agree is mental privacy. A central question in current debates about this right is the following: does mental information inferred from brain data need special protection that distinguishes it from other types of personal data? In his article, Wajnerman Paz defends an affirmative answer to this question based on the idea that mental privacy is a psychological capacity and that this capacity is intimately connected with the narrative and relational conception of personal identity.

Inglese and Lavazza's paper expresses one of the most disturbing questions for neurorights: What are we going to do with people who do not want to be protected from the advances of neurotechnologies? The debate is not minor and represents an inescapable practical challenge in a posthumanist scenario, every time less distant from reality.

Borbón and Borbón's paper questions the relevance of including free will as a specific neuroright, since this concept is so elusive and has been unsolved for at least 2,000 years. Another interesting aspect in this article is that it cares about unequal and plundered regions of the planet such as Latin America. In this regard, the authors point out that the right to equal access to mental augmentation could be considered inappropriate “in developing countries, such as Latin American ones, as some of them cannot even provide access to the most basic needs, such as nutrition or health care, and the guarantee of human rights.” Moreover, this article recalls the need to take into serious consideration the cultural aspects of several world regions, not only the Anglo-Saxon or “developed world” perspective. Authors such as Karen Herrera-Ferrá, rightly insist on integrating this issue into the mainstream debate (see Herrera-Ferrá et al., 2020). In sum, this document asks whether neurorights are actually necessary and points out the following: “We view with skeptical eyes the advisability of creating a new category of rights.”

The paper by Larrivee provides a philosophical analysis of human-machine relationships. Although his article does not directly deal with the concept of neurorights, it does offer a perspective that can inform in a relevant way the ethical-legal debates about the values and principles that should guide the development of these rights. In response to proposals for an ontological and ethical parity between machines and humans, Larrivee defends the idea that there is a dynamic, operational, global integration in living beings, confirmed by neuroscience, which “offers a basis for ascribing ontological distinction to humans and for informing ethical values guiding human-machine relations.”

In sum, both neurolaw and neurorights are central notions that cannot be ignored. An in-depth analysis of these conceptual challenges involves difficult practical tasks, but it is urgent. We must also think about the near future and prepare the lawyers of the coming decades. They should be able to deal with these phenomena with greater specific knowledge. To do this, one important working path is to update the syllabi of law schools by including the subject of neurolaw. This could provide students with the ability and the conceptual and interdisciplinary tools to adequately address these novel issues in the future.

## Author Contributions

All authors listed have made a substantial, direct, and intellectual contribution to the work and approved it for publication.

## Conflict of Interest

The authors declare that the research was conducted in the absence of any commercial or financial relationships that could be construed as a potential conflict of interest.

## Publisher's Note

All claims expressed in this article are solely those of the authors and do not necessarily represent those of their affiliated organizations, or those of the publisher, the editors and the reviewers. Any product that may be evaluated in this article, or claim that may be made by its manufacturer, is not guaranteed or endorsed by the publisher.
